# The missing bit in the middle: Implementation of the Nationals Health Services Standards for Papua New Guinea

**DOI:** 10.1371/journal.pone.0266931

**Published:** 2022-06-24

**Authors:** Elisabeth Schuele, Colin MacDougall

**Affiliations:** 1 Department of Public Health Leadership and Training, Faculty of Medicine and Health Sciences, Divine Word University, Madang, Papua New Guinea; 2 College of Medicine and Public Health, Flinders University, Adelaide, Australia; Deakin University, AUSTRALIA

## Abstract

**Objective:**

This case study examined implementation of the National Health Services Standards (NHSSs) as a continuous quality improvement (CQI) process at three church-based health facilities in Papua New Guinea. This process was designed to improve quality of care and accredit the level three health centers to level four as district hospitals to provide a higher level of care. The aims of the paper are to critically examine driving and restraining forces in CQI implementation and analyses how power influences agenda setting for change.

**Methods:**

Semi-structured interviews were conducted with nine managers and eight health workers as well as three focus group discussions with health workers from three rural church-based health facilities in Morobe and Madang provinces. They included senior, mid-level and frontline managers and medical doctors, health extension officers, nursing officers and community health workers. Thematic analysis was used as an inductive and deductive process in which applied force field analysis, leadership-member exchange (LMX) theory and agenda setting was applied.

**Results:**

Qualitative analysis showed how internal and external factors created urgency for change. The CQI process was designed as a collective process. Power relations operated at and between various levels: the facilities, which supported or undermined the change process; between management whereby the national management supported the quality improvement agenda, but the regional management exercised positional power in form of inaction. Theoretical analysis identified the ‘missing bit in the middle’ shaped by policy actors who exercise power over policy formulation and constrained financial and technical resources. Analysis revealed how to reduce restraining forces and build on driving forces to establish a new equilibrium.

**Conclusion:**

Multiple theories contributed to the analysis showing how to resolve problematic power relations by building high-quality, effective communication of senior leadership with mid-level management and reactivated broad collaborative processes at the health facilities. Addressing the ‘missing bit in the middle’ by agenda setting can improve implementation of the NHSSs as a quality improvement process. The paper concludes with learning for policy makers, managers and health workers by highlighting to pay close attention to institutional power dynamics and practices.

## Introduction

Effective health policy implementation to improve the quality of service delivery for better health outcomes is a major issue in many low- and middle income countries (LMIC) including Papua New Guinea [1, 2]. In LMICs more than 8 million people die annually from illnesses treatable by health care providers; with 60% related to low quality care and 40% low use of health care services [3]. Current research shows that improvements in health outcomes will continue to be constrained if the quality of care remains weak [3]. Governments therefore need to provide leadership in developing national health policies. However, as policy actors deal with conflicting options, power shapes their actions and choices concerning the policy agenda to improve quality of care [4].

Quality health provision includes service implementation strategies [3, 5], but the gap between knowledge and implementation is widely recognized as a whole health system challenge [6, 7]. This gap, and its effect on health policy implementation, also influences power relations experienced by both health workers and managers [6]. Power shapes social interactions and is present in actions, inactions, processes of policy implementation and outcome of quality health services [8].

There are many interconnected reasons for Papua New Guinea’s (PNG) health service quality remaining sub-optimal [9]. Factors contributing to the continuous struggle to delivering quality health services include: mountainous terrain, poor transport and other infrastructure systems, limited financial investment, ongoing law and order problems, and deteriorating rural health facility deficiencies compounded by severe shortages of human resources for health. In addition, frequent stock-outs of essential medicines and delay in the provision of funds to the provinces and lower-level health services contribute to poor health services [10]. This is demonstrated by problems such the measles outbreaks from 2013 to 2015 [9], increasing multi-drug resistant tuberculosis [11, 12], and the highest maternal mortality rate in the region of 215 per 100,000 live births [13].

PNG’s National Health Plan (NHP) 2011–2020 and its National Health Service Standards (NHSSs) were developed to improve service delivery and health outcomes [14]. The NHSSs potentially provides an important framework to “promote continuous quality improvement” as a crucial process to improve the quality of health services in the country [15: p.6].

Although factors driving quality are partly understood [2] many problems influencing policy implementation remain unsolved, highlighting a need to deepen understanding of driving and restraining forces to implementation of the NHSSs [16]. In particular, Volume 2 of the NHSS three-volume specifies the standards and measurement criteria to be used for accreditation of health service delivery facilities. However, these quality standards have not been widely implemented because of the complexity of detail and missing technical advice for operationalising local implementation [9]. The gap between policy design, implementation guidelines and its implementation, the ‘missing middle’, persists. This potentially has serious consequences for health outcomes [1, 17].

### Papua New Guinea’s health system

PNG has a decentralized health system based on a Primary Health Care (PHC) approach. The health system consists of seven level of health services as a network of aid posts, community health posts, health centers, district hospitals, provincial hospitals, regional hospitals and one national hospital [9, 18]. Healthcare is delivered through a combination of government and church-based health services as well as some private health facilities. It is estimated that church-based health services contribute almost half of primary health services, especially in rural and remote areas [19].

In 1995, an Organic Law on Provincial and Local Level Government (OLPGLLG) was enacted, changing the management structure for health care delivery, and transferring authority over funding and service delivery responsibilities to provinces and newly-created local level governments [20, 21]. The National Health Authority Act 1997 established the legal framework of health boards to advise on the functions of various level of government in health services delivery, including introduction of the National Health Services Standards (NHSSs) and coordination of NHP implementation [9, 20].

The most recent reforms involved further decentralization and devolution of power by establishing Provincial Health Authorities (PHA) and District Development Authorities (DDA) to provide the functions and responsibilities of service delivery by district administration [9, 22].

### Church-based health services

The Church Health Services (CHS) umbrella organization is a key partner in the implementation of the NHP. CHS is an indispensable partner to the National Department of Health (NDoH) since it plays a key role in filling the gap left by the Government in health service provision, especially in rural and hard-to-reach areas [23]. CHS is responsible for managing church-health service grants provided by the NDoH to the church agency members and ensures the effective and efficient use of financial resources and exercising financial control over the received grant [24]. In April 2015, the national office of one CHS member agency initiated a continuous quality improvement (CQI) process to both improve quality of care and accredit the level three-health centers to level four as district hospitals to provide a higher level of health services. CQI involved a quality assurance at three health centers as a baseline and provided a set of priorities of where and how to start the implementation process of the NHSSs. The quality improvement process method was based on the plan-do-study-act cycle (PDSA), which started with planning and implementation of small-scale interventions, assessing the progress and outcome for change and lessons learned informed action of the next cycle [25].

Force field analysis, derived from Kurt Lewin’s force field theory, is used as a technique for planning and implementing changes in organizations [16]. The force field analysis provides a framework to examine the driving and restraining forces in the implementation process of the NHSSs [26]. This theory shows how the interplay of these forces results in the equilibrium that may increase change, while reducing forces against change increases pressure for change in implementing the NHSSs [16, 27]. Force field analysis enables to uncover hidden power relations within and between structures of an organization by challenging current patterns and developing new approaches and structures to implement the quality standards [27, 28].

This research was designed to examine the implementation of the NHSSs as a continuous quality improvement (CQI) process at three church-based health facilities operating under a church-health services governing structure in PNG. The main aims were: to critically examine driving and restraining forces in the implementation process of the NHSSs; understand how hidden power relations work in the implementation process; and assess agenda setting to influence change.

## Materials and methods

The epistemological foundation of this qualitative case study is informed by constructivism and critical theory [29]. Combining constructivism, critical theory and force field analysis allows researchers to better understand and interpret how the CQI agenda as an action for change process contributed to the implementation of the NHSSs [29, 30].

This case study was designed to listen to the accounts of health workers and managers at different levels of a CHS agency to answer the research objective. Force field analysis (FFA) is one of the forerunners of Action Research and provides a theoretical perspective from which to frame research questions, and analytical principles to underpin coding and analysis of results. It is therefore highly appropriate to understand and analyse the three stages in which organizational change unfolds [30]. In the first stage, unfreezing, staff revealed the current practices and how the vision for change was created. The moving stage involved the developing of new approaches and structures and by refreezing new routines and arrangements are established to achieve the desired goal [28]. Of particular interest to this study is the way in which FFA directs our focus to developing a theory or theories of change to understand the unfreezing, change and refreezing process.

At the same time, critical inquiry allows the articulation of power relations within and between the organizational structures [29] and how driving and restraining forces impact on effective CQI implementation in three health centers of a CHS agency located in Madang and Morobe Provinces. To unpack these power relations, leader-member exchange (LMX) theory is applied to conceptualize leadership as a process on the interaction between managers and health workforce [31].

Data were collected in two rural health centers of Madang province, one in Madang district and one in Sumkart district on Karkar island. In Morobe province one rural health center at the coast of Finschhafen district was included in the study. The data collection was conducted by the first author together with two male colleagues who hold master’s and doctoral qualifications, are fluent in the local language and have extensive qualitative data collection experience. The first author knew some of the participants through previous public health consultancy work with a different organization seven years ago.

### Participants and procedures

The sampling for the interviews and focus group discussions (FGDs) was purposive with the aim to consider individuals working at the agency national office, two regional offices and three level three-health centers [32]. The accounts of the health workforce within a CHS member agency to a CQI implementation process were used to identify hidden power dynamics working within and between management and organisational structures (See S1 and S2 Files).

Semi-structured interviews with 17 health workers and managers and three focus group discussions (FGD) were conducted. 19 women and 28 men were included from a variety of settings and were employed in a range of roles, including facility, frontline health workers, and senior, mid-level and frontline managers. Participant details are summarized in Table 1.

**Table 1:** Distribution of study participants based on sex.

	N	Women	Men
**Focus Group Discussions (FGD)**			
FGD1	5	2	3
FGD2	9	5	4
FGD3	8	2	6
Total	22	9	13
**Semi-structured in-depth interviews**			
Health Work Force	8	4	4
Clinicians	2	0	2
Nurses/Midwives	6	4	2
Managers	9	2	7
Rural health facility	4	0	4
Regional office	2	0	2
National office	3	2	1

An interview guide with open-ended questions included prompts reflecting on the research questions. During the course of the research, additional questions and themes were explored based on the responses of participants. The interviews and FGDs lasted 30 to 60 minutes and were conducted between September 2018 and May 2019. Participation was voluntary and they provided written consent at the time of interviews and FGDs. Ethical approval was obtained by the university research committee (UREC) and the CHS agency.

### Data analysis

Interviews and focus groups were audio recorded and transcribed verbatim. One interview and one FGD in Tok Pisin was transcribed and translated into English by a Papua New Guinean.

QDA Miner (LITE v2.0.5.) software was used to assist with coding and analysis of the data according to the thematic framework. The thematic analysis as an inductive and deductive process started with transcripts of the first interviews to explore the topics and concepts from the interview data [33, 34]. Pseudonyms were assigned to participants in transcripts and the presentation of the findings to hide identities. In the results section, we explained how theory guided the coding and analysis.

The analysis process comprised of the development, revision and comparison of codes, which resulted in a coding structure of the sub-themes and themes, mapping and interpretation of the data [33]. Field notes and informal discussion records were reviewed and used to triangulate the data. Two researchers initially coded the transcripts with the first author developing the codebook with themes emerging from the data. These themes were discussed and revised in team discussions and consensus developed on the coding structure. The final stage of the analysis involved further interpretation of the findings and the interaction of themes and sub-themes as they related to the study aims.

In presenting the findings, participants’ quotes are used from a broad range of interviews to demonstrate trustworthiness of interpretation and evidence for interpretative rigour [35].

## Results

We analyzed the results by first translating the components of FFA into codes for enablers, barriers, revision for change, and ideas for the theory of change involving unfreezing and refreezing. Next, we examined data closely to understand the elements of change and decided that LMX theory functioned well to explain and predict in the most parsimonious way. As a result, we translated the key elements of LMX into codes and applied them to the data. Finally, we combined these two theories with relevant evidence to organize the results section so it draws on relevant theory and literature.

### Creating a vision for change

A key first step in FFA is to define clearly the vision to drive change. Internal factors that created organizational readiness was the widespread dissatisfaction in the quality of health service provision expressed by both senior management and frontline health workers. National senior managers recognized the poor quality of service provision and by demonstrating a commitment to quality, acknowledged an urgent need for change. This stimulus to change was based on the realization expressed by a senior manager that “…even our behavior, how we deal with patients, our commitment, we say we are committed, we are saying we are providing better services but these things are slowly, gradually falling apart”. He expresses his dissatisfaction with the current performance levels at the facilities and searched for a way to start a continuous quality improvement process.

Interview transcripts showed a widespread recognition for the urgent need to improve quality of health service provision. A midlevel manager observed that “the quality of services provided have declined over the years” (John/midlevel manager).

There was a general agreement between all participants that the introduction of quality improvement was ‘over-due’ (Douglas/frontline health worker) and the CQI process will improve patient care since “our clients should get a better treatment” (Chris/health worker).

Moreover, there seems to be a realization that quality improvement measures contribute to health workers own health and safety. As a male clinical staff highlighted:

“Improving the standards is for the benefit of our patients. That is the whole idea, but also staff will benefit. Good surrounding, good infection control measures. Then it is protective to the staff as well. Everyone will benefit, staff, patients, the management for their reputation” (Mark/health worker).

An external impetus for change was the vision for accreditation of the health centers, commonly called ‘hospitals’, to district hospitals.

Most respondents expressed the prospect of accreditation as a motivating factor to engage in the quality improvement process.

“Our idea is to get to the next level, which is a level four hospital! So, we are trying our best to meet the standards. That motivates us to work towards changes” (Chris/health worker).

Quality control officers were engaged to lead the change efforts and made responsible to introduce the NHSSs to the staff at the health facilities. This was the time for a number of participants to familiarize themselves with the NHSSs. A frontline manager and a male nurse explained this as follows:

“…I have heard about them but actually I did not involve and implemented them and I did not see the importance of it. But when it was introduced, during that time I came to realize the importance of the health services standards” (Bruno/frontline manager)

“I worked for a couple of years. Maybe around ten years, but I’ve never heard of these standards” (Chris/health worker).

### Change—Participation in the collective process

Once the analysis has articulated a clear vision for change, Force Field Analysis requires a theory of how change occurs. However, because a theory of change is not built in to Force Field Analysis, we derived interlocking theories of change from the data. Senior management introduced quality improvement as an empowerment process in which leaders and the health workforce at the health facilities were invited to a collective process.

Most participants in interviews and FGDs appreciated the adoption of a participatory action research framework in which all facility staff, clinical, management and support personnel alike were invited for bi-annual planning and review meetings and the plan-do-study-act cycle was applied [36, 37]. Rhonda, a senior manager explained the start of the intervention, “we conducted meetings and every staff from each area of work was invited to participate and give their views”.

The collective, reflective process was designed and linked to implementing of facility action plans. Staff were divided into three groups to assess a particular area. Groups presented their observations, the different issues were ranked and an action plan developed. A female and male manager illustrated the process:

“They go and share in groups, assess themselves…They have to rate themselves… They go around, check carefully against the checklist. They give themselves a rating of each item in the action plan that they go through… An all other staff in the other groups have to agree if the rating is correct … So we had participation of staff from all areas”. (Rhonda/senior management)

The participatory action research framework encouraged teamwork as a new way of working together. Participants in both interviews and focus groups commented on the importance of teamwork as Helen, a nurse stated, “if anything, we work together!”

The quality assurance officers played a key role in involving staff and dividing the different tasks. This supported collegial networks. In one facility, Douglas, a nurse, appreciated their working relationship and described it as follows:

“Actually, I was appointed as the team leader in the quality assurance process. All of us staff are involved. We have officers that work on the standards, see that the activities are carried out. So we have teams”.

Bruno, a frontline manager summarized the collective and reflective process as, “we have our plan and we have our meetings and we have our assessment, and we have our committee. These is the internal support that we have which is very important for the work”.

#### Power relations–driving and restraining forces at the facility level

Power as a component of a complex social system ensures an asymmetrical system of relationships [38]. The implementation of the CQI process caused unanticipated behavior patterns of people and uncovered power dynamics within the health facilities and between management levels. Different forms of hidden power relations were revealed as driving or restraining forces that supported or caused resistance to implementation of the quality improvement agenda.

The results suggests that there is an in-group and out group based on participation in the CQI implementation process [31]. Not only the quality assurance officers, but also other staff, drove implementation. They worked with the senior management and as such formed the in-group [31] and took on additional responsibilities that recognized them as ‘champions’. Mark described the in-group as follows:

“We have some people who are passionate about employing the standards. So, they provide a local push factor. They were planning meetings, they organized the staff, they improved certain area which needed it especially those standards that wouldn’t need money to improve it” (Mark/health worker).

However, many participants indicated resistance to the newly introduced process thus practising power. Their relationship to senior management was less attuned than members of the in-group [31]. Andrew, a frontline manager at a facility justified his reluctance to participate as follows:

“It was not planned from bottom up, but from top down. So it worked for some time but then eventually stopped”.

The out-group was characterized by Rhonda, a senior manager as,”staff have been in the systems too long”, and their attitudes obstructed the implementation process.

While the relationship of the out-group to senior management was constrained, it also affected the relationship to other staff who were actively involved in the implementation process in a negative way. A number of participants formulated their frustrations concerning the out-groups which included clinical, administrative and support staff who exercised power by demonstrating resistance to change through non-participation.

Despite the broad participation in the initial planning and review meetings, the relationship between the in-groups and out-groups resulted in the reduction of participating staff in the implementation of the quality improvement agenda. Bruno, a frontline manager concluded:

“Only a few staff were involved and they wanted the standards to be improved. But when there are only a few staff showing interest and others not, it is like pulling a very heavy bag and the progress will be slow”.

#### Leadership and power relations at the facility level

One emerging theme was the perception of leadership styles, the tension between bottom up and top down leadership at various levels to support the change process [39]. Senior managers at the national office expected a bottom-up approach to leadership and monitoring of the CQI agenda.

Senior management encouraged a dyadic relationship with the health facilities management and expected collaboration, taking on more roles and responsibilities in driving the quality improvement process. A senior manager pointed to the management at the facilities stating that, “they have to be proactive. They have to initiate and take responsibility” (David/senior manager).

However, not all of the facilities developed a leadership and monitoring structure pointing towards non-linearity of a complex interrelated system in which power is exercised by manager at the facility level. Some participants experienced a sense of poor leadership as a restraining force.

In one facility, participants of a focus group complained their quality control officer “is wearing too many hats. He is not focusing on the quality work anymore” (FDG 3). They requested for another health worker to be assigned to move the process.

At another facility, a female nurse explained that the quality control officer is leading the committee. Meetings are conducted, internal assessments carried out and staff instructed what areas need improvement (Helen/health worker). However, a restraining force in the implementation was the lack of monitoring mechanisms as Stella, a nurse from the same facility pointed out:

“….yes, we used to conduct them last year, but this year we didn’t conduct any. There is no one actually monitoring the process and that is still a problem”.

A driving force emerged through two female nurses in charge who explained that they have taken on the leadership responsibility of monitoring the implementation of standards on their wards.

Within the complexity of power relationship as driving and constraining forces leadership emerged and a nonlinear monitoring structure that contributed to the quality improvement process. Rhonda, a senior manager confirmed that reports have been submitted regularly to the regional and national management level and funding made available to implement some of the action plan activities.

#### Leadership and power relation at management levels

Leadership support from all organizational levels is a critical element for effective change at the facility level [40]. Practices of power as a significant restraining force were exercised by mid-level and frontline managers. There is a general recognition from participants of both the national office and the health facilities that insufficient support is provided by the facility administrations and the regional offices which slowed down the CQI implementation process.

A major constraining factor at the facilities was the administration’s decision-making power over financial resources by the administration. Although improvement in hygiene and sanitation has been regarded as a change, two female nurses raised concerns about the inconsistent supply of simple cleaning materials such as detergents, soap and brush. Stella, a nurse explained:

“I think there is still a problem of purchasing new materials, like especially mops and detergents for daily use; this is still a big problem”.

Fiona, a midwife, pointed out that due to the very old vehicle the family health team is unable to reach the mountain villages of the catchment area to provide the expanded program on immunization (EPI) and antenatal care. Women are expected to come to the hospital to access theses services.

The regional offices were identified as an opposing force to change by maintaining the status quo. A few participants pointed out the important role the regional offices play since they are the link between the facilities and national office as well as the provincial government. The need to cooperate with the government at provincial level was explained by Monica, a senior manager but “the provincial [government] health offices are not approached. The regional secretaries are responsible for this”.

The disappointment for the lack of regional commitment–the missing middle to provide leadership support and guidance was expressed by participants working at all facilities. One response by a male health worker indicated that there is “no push from the management”. He continued explaining “the regional office didn’t really show support” and did not show interest in “even simple things like turning up for a meeting or when we call” (Ted/health worker).

Regional managers demonstrated a frozen relationship the workforce at the facilities. While staff at the facilities expected leadership support in planning and monitoring of the quality improvement process by the regional managers, they exercised power by non-participation. A male frontline manager formulated his disappointment as “they are not coming and see how well, or what has been done” and feels that “they are not talking it seriously and the plan will be shelved away and the dust will cover it” (Gabriel/frontline manager).

### Power over resources

#### Translating policy into practice

The NDoH holds the power over policy formulation. A challenge in implementing the NHSSs is that power can manifest itself in the way the policy is formulated. It became clear that understanding the complex NHSS document is a key in translating the document into practice as a CQI process. Participants critically commented on the NHSSs document. All participants in interviews and focus groups expressed that the document is not user-friendly because “it’s very technical and not as easy for simple people to understand what the standards are” (Monica/senior manager). John, a midlevel manager admitted that “I can’t fully understand it”. Mark, a health worker summarized the discussion concerning the language of the very long document as follows:

“I have read it over and over again trying to grasp the basic concept. Although it is outlined in detail, the main points are not really highlighted so that we can understand it better”.

Focus group participants pointed out that they have not seen the NHSSs document since only some of the management team received a hard copy. Although the document can be downloaded, a male health worker pointed out the difficulties of internet access in rural areas.

Although successful accreditation as district hospitals is based on the NHSSs, implementation guidelines have not been on the agenda of the NDoH and identified by participants as a missing link in the policy transfer process. A few participants explained that reading the document is not the only issue “but actual implementing it is the problem” (Chris/health worker) since there “was never a clear implementation guideline” (Monica/senior manager). Many participants felt the lack of such guidelines is a major constraining force in the implementation process.

“I read the NHSS document. How to implement it, there was never a clear guideline. So we have to work on our own how to get this done at the facility level” (Rhonda/senior manager).

#### Power over financial, technical and human resources

Some participants pointed to the power of the NDoH over financial and technical resources. They spoke about the importance of an implementation system and supportive mechanisms by the NDoH, which includes financial support. Ted, a health worker highlighted appropriate resource allocation by NDoH has been off the policy agenda.

“You [the NDoH] created the standards without funds, without support, without help in the process. And if there are funds available you have to make it known to the management”.

In addition to the availability of financial support, participants expressed the missing technical support by the NDoH. Managers and health workers felt that the NDoH is responsible for explaining the various standards and “this are the things you must meet, and then this is the process” (Mark/health worker).

A true constraining factor in the CQI process is the availability of human resources for health.

An adequate number and appropriate role delineation of the health workforce is crucial for driving health service performance. Although the facilities provide services as district hospitals, the number of staff are calculated according to level-3 health centers. A severe shortage can be observed especially at the weekends and at night when only a few health workers are on duty and responsible for all the wards. The discussion was summarized by Andrew, a frontline manager as follows:

“We consider ourselves as a rural hospital but we have only 34 officers. Compared to the standards, rural hospitals should not have only 34 staff. So the implementation of the standards is difficult because of things like this”.

## Discussion

This study enabled an exploration of accounts by managers and frontline practitioners of the quality improvement agenda in implementing the NHSSs. Force field analysis was used to reveal driving and restraining forces in the change process of the CQI process. Using critical theory coupled with leader-member exchange (LMX) theory allowed an understanding of how power operates within the system in which the intervention was implemented [29, 31].

### How to reduce restraining forces and build on driving forces

In this section we use theory to understand how to change restraining and driving forces to establish a new equilibrium. Some of the front-line managers, support staff and health workers exercised power through inaction resisting the collective effort to implementing change. They used their discretionary power displayed by withdrawing their involvement in the CQI implementation process [41]. While this was an act of resistance to change, our analysis suggested they comprised an out-group pointing to low-quality relationship with senior management [42]. This led us to apply leader-member exchange (LMX) theory, which is centered around dyadic relationships between leaders and followers. LMX theory explains how particular leadership practices defined *in groups* and *out groups* among followers. In group members are more highly valued by leaders and receive more encouragement and opportunity, contrasting with *out groups* who receive less attention which often causes them to perform their roles and more basic ways and resist change [31]. The application of LMX theory explained why health workers who were part of *out groups*, experienced a slow implementation of quality improvement activities. It became apparent that the effective management of this ‘implementation gap’ [6: p.63] has not been considered by senior management. Effective change and implementation would therefore be enhanced by leadership that includes and rewards workers to minimize the formation and perpetuation or out groups who will have trouble contributing to change.

Leadership commitment to the quality improvement agenda involves all levels of the organization [40]. Regional managers located strategically between the national top managers and the frontline personnel have a crucial role in filling “structural holes”, the missing bit in the middle of the organization [43: p.4]. The findings suggest regional managers’ exercised positional power by resisting the new approach of the CQI process thus contributing to the policy implementation gap [6, 44]. These complex, interrelated mechanisms of power relations exist at “the edge of chaos” [45: p.396] as a major restraining force in form of inaction that influenced the implementation of the NHSS policy document at facility level [46].

Policy actors at the NDoH have the power to influence the policy agenda setting and formulation of the NHSSs [4]. Participants view the language of the NHSSs document difficult to interpret without detailed regulations or implementation guidelines that facilitate implementation at the facility level [9, 47].

Power relations operate between the NDoH and the CHS agency providing services [48]. Participants addressed the power over resources by policy actors at the NDoH, which negatively impacted the CQI process. This includes an insufficient number of human resources for health in the facilities, lack of financial resources, the absence of technical support and provision of guidelines to implementing the NHSSs. These constraining factors are common in other low-and middle- income countries compared to high-income settings [49].

The effect of power is recognized as the heart of policy agenda setting [4, 50]. The findings reveal the ‘missing bit in the middle’ between policy formulation and implementation [51]. The degree of significance of the missing support by the NDoH in developing implementing strategies to quality improvement, thus exercising power, is an important finding of this study. Kruk, et al. [3] emphasize quality improvement in complex, interconnected health system require not only micro-level changes, but “structural reforms that act on the foundations of the system” [p.1197].

System-wide strategies to strengthening governance and coordination include the availability of resources and monitoring performance as a part of improving quality of health services [3]. This study suggests the impact of contextual factors to the implementing trajectory of the NHSSs are remarkable and reflected in participants accounts of ‘*so we have to work on our own how to get this done’*.

### Understanding current patterns–the case for change

**Fig 1:**
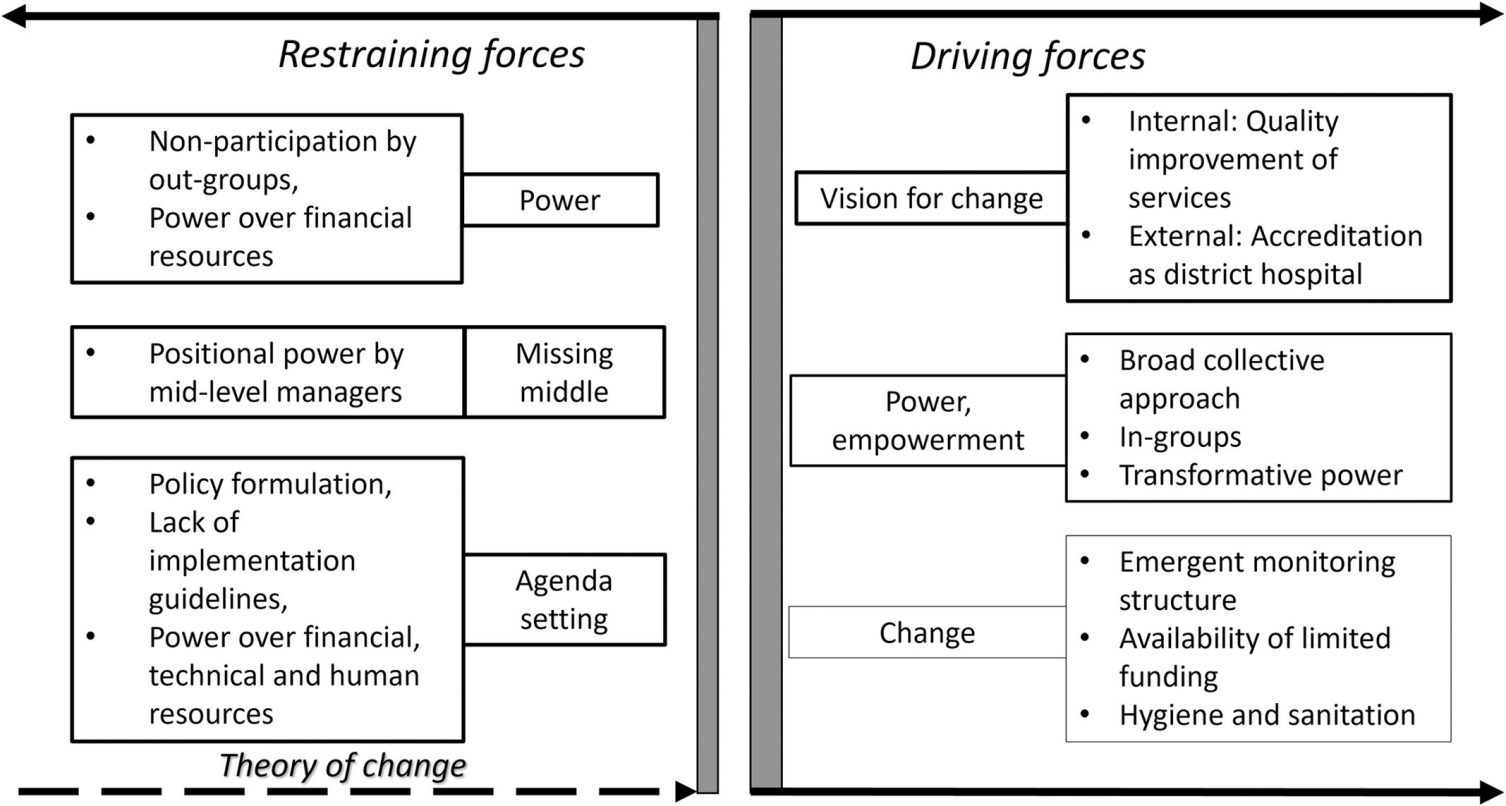
Restraining and driving forces. The above figure indicates the restraining and driving forces of the National Health Services Standards implementation process. On the left there are set of restraining forces and on the right are the set of driving forces. The two vertical lines in the middle demonstrate the gap created by the opposite directions of the forces.

Fig 1 presents those restraining and driving forces of the current situation that form a theory of change to achieve a new equilibrium. The size of the shape outlined represents the influence of the theme to implementing the NHSSs. While dealing with the restraining forces, attentions need to be paid to the driving forces. Unfreezing involves moving the restraining forces of the left-hand side towards the middle and making a case for change by maximizing the driving forces [27].

The organizational readiness for change, as a major driving force, was identified by all participants who expressed the need and beneficence for change [52, 53]. It paints a picture of how unfreezing, the goal for change was related to both internal and external driving forces—improved quality of health services and the prospect of the facilities being accredited as district hospitals.

The study provides an example of producing a collaborative process by actively involving the different types of personnel at the health facilities: from the doctors to the Community Health Workers, the administrators and the support staff. The finding builds on LMX theory whereby a collaborative action and reflection cycle was applied that created high-quality relational power mechanisms that operate in the interaction between leaders and the health workforce [30, 54]. That evidence suggests that the level of satisfaction in the supervisor—subordinate relationship influences subordinates experience of access to resources and information and consequently perceive themselves as empowered [54]. It recognized a complex social change process and created actions and motivation of the quality improvement process [55].

Uncovering power dynamics within the health facilities are part of participatory approaches [56]. In all facilities, power relations as a driving force, were recognized through individual engagement by health workers who took on additional responsibilities, which contributed positive to the quality improvement process [6]. In agreement with Gardner, et al. [36] findings in a study in Australia, the motivation of continuous engagement in the quality improvement process can be attributed to individual champions. These champions formed the in-groups around them marked by high quality leader-member relationships, empowered to drive the CQI agenda.

While the quality improvement process is implemented within complex interactions and power relations act in a non-linear way, leadership requires holding people together “to reflect and make sense of what has happened” [57: p.48].

Leadership at the facilities emerged from the bottom up in which monitoring networks appeared to facilitate change as an emergent property of self-organization [45, 46]. These efforts of self-organization were supported by the national office in provision of resources to improve the waste management system and supply of important equipment for the facilities. Their practice of power that drove the quality improvement agenda was transformative–demonstrating ‘power with’ rather than ‘power over’ [58: p.1].

The findings of this case study demonstrated how forces-field analysis was used to identify restraining and driving forces in the NHSSs implementation process by conceptualizing change as unfreezing, change and refreezing [27]. The proposed change happens by applying the LMX theory to activate the ‘missing bit in the middle’ and mobilizing power relations as an empowerment process.

**Fig 2:**
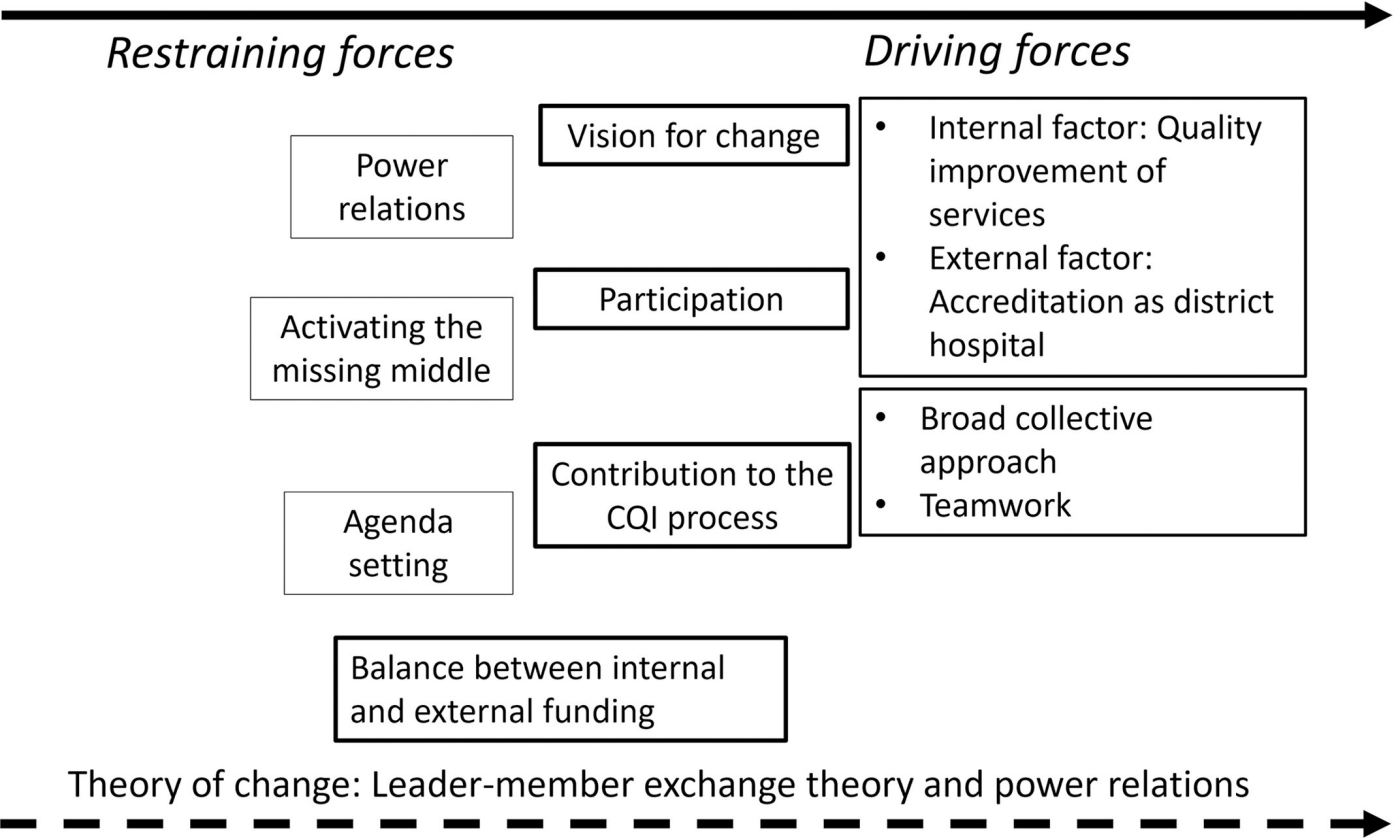
Theory of change. The above figure demonstrates that restraining forces moving towards the direction of driving forces by using leader-member exchange theory. It also deals with power relations to enable the change in the implementation of National Health Services Standards.

Fig 2 draws on LMX theory and shows that despite the powerful vision for change and the broad involvement of the workforce at the beginning of the CQI process some health worker and management staff formed out-groups and exercised power through non-participation. Based on the LMX theory the broad collaborative CQI process can be reactivated so that *out* groups exercising discretionary power are reduced, and the health workforce is motivated and respected for their contribution in the process [31, 59]. This will be part of a new strategy that coupled with the availability of sufficient financial resources reduces restraining forces against change [16, 27].

Second, internal change can happen by activating the ‘missing bit in the middle’ in reducing positional power displayed by mid-level management and for senior management to develop high-quality exchange with them [31, 59]. Senior management is in the position to act on theses restraining forces and foster productive power relations [50].

Third, activating the ‘missing bit in the middle’ points towards using power of influence by the NDoH in reducing the gap of NHSSs formulation and implementation. Policy implementation mechanisms and accountability strategies should be developed in partnership with the implementing governmental- and church-based agencies [3]. Reducing the ‘missing bit in the middle’ means for the policy actors to use power to act by taking on board the findings of this case study.

### Justification for the study with strengths and limitations

The justification of, and strength of, this case study was the use of multiple theories in data analysis. This allowed for themes to emerge from the FFA that provided a rich understanding of enabling and restraining forces of a CQI process. The use of FFA as a dynamic change management instrument confirms Swanson & Creed’s [60] conclusion that while change does not happen quickly, it can be planned and managed by understanding the dynamics of the complex interrelation of forces in different contexts.

FFA supported the use of LMX theory to investigate power relations evident through *in* and *out* groups and the ‘missing bit in the middle’. The findings suggest lessons for moving the change process forward by the importance of communication and high-quality exchanges between the leaders at different levels [31].

The application of agenda setting theory revealed that the use of power has resulted in holding back change by avoiding addressing the ‘missing bit in the middle’ in the implementation of the NHSSs. A theory of change is presented by applying multiple theories for change to happen to reach the desired outcome in successfully implementing the NHSSs.

A limitation of this study is that the data were derived from only three facilities of one CHS member agency.

## Conclusion

The importance of creating organizational urgency for change as a collaborative process, encouraging a broad participation of all clinical, management and support staff, was identified.

The study uncovered power relations within the organisation exercised by front-line health providers and managers at different levels, influencing the implementation of the CQI process in both positive and negative non-linear ways, either narrowing or widening the NHSSs implementation gap.

Leadership requires an organisation-wide commitment to implement the NHSSs as a CQI process [36]. Leadership at all levels is vital for dealing with change process complexities and power relations, to ensure that changes in the way of working become part of routine service quality improvement. Our study findings reveal through resistance and inaction practice positional power mid-level and frontline managers hinder quality improvement and reflective practice as a monitoring strategy in the implementing process of the NHSSs.

Contextual elements further complicate the complex power dynamics of the NHSSs implementation process for health service quality improvement in PNG. The language of the document itself and the lack of effective implementation strategies by the NDoH contribute significantly to the implementation gap. These are clear recommendations for policy makers coupled with the need to provide sufficient financial and human resources as well as a technical support system as a whole system approach [61].

Given that this study generated relevant information and highlighted dimensions of power dynamics and practices, paying close attention to further improvement of implementing the NHSSs as a quality improvement process is crucial for policy makers, managers and health workers to help to build the ‘missing bit in the middle’.

## Supporting information

S1 File Semi-structured interview guide. (DOCX)

S2 File FGD question guide. (DOCX)
