# Supplementary material for: The missing bit in the middle: Implementation of the Nationals Health Services Standards for Papua New Guinea
Source: PLoS One. 2022 Jun 24;17(6):e0266931. doi: 10.1371/journal.pone.0266931 (PMC9231790; doi:10.1371/journal.pone.0266931)
Supplement: S1 File — (DOCX) [file pone.0266931.s001.docx]

**S1. Semi-structured Interview Guide**

- How were the NHSSs (Quality assurance process) introduced in this facility? (probe on when the implementation process started, what was known before about the NHSSs, feeling about the introduction of the NHSSs/Quality Assurance process)
- What is your experience in implementing the NHSSs (Quality assurance activities)? (probe on his/her role, when the process started, why did it start)
- What parts (standards) of the NHSSs are you implementing? (Why? How? Who is involved?)

Rephrase: what quality assurance activities are you implementing?

Probe on the implementation process.

- How user-friendly is the NHSSs document? (Probe on practicality and translation into practice).
- What support do you have/did you receive to assist in the implementation process probe on various levels: hospital level, regional level, Lutheran Health Services national level, community, Local Level Government, NDoH, Province)

(Probe on availability of various clinical guidelines, use of guidelines, supervision checklist by NDoH.

Probe on LHS: workshops, trainings, meetings, supervision checklist, etc.)

- What changes did you experience? (probe on perceived enablers, changes at different levels, national, regional, facility level and self) How do you feel about it?
- What barriers did you encounter? (probe on barriers at different levels) How do you feel about it?
- How do you monitor the implementation process of your plans? Who is involved?
- How can the implementation process move forward? (probe: self, at different levels of LHS management: facility, regional, national, level, and government – Provincial Health Authority, NDoH)

**Additional question for quality assurance officers:**

- How was the committee formed? (probe on selection criteria, roles of committee members, duration of meetings)
