# Supplementary material for: The missing bit in the middle: Implementation of the Nationals Health Services Standards for Papua New Guinea
Source: PLoS One. 2022 Jun 24;17(6):e0266931. doi: 10.1371/journal.pone.0266931 (PMC9231790; doi:10.1371/journal.pone.0266931)
Supplement: S2 File — (DOCX) [file pone.0266931.s002.docx]

**S2. FGD Question Guide**

- How were the NHSSs introduced in this facility? (probe on when the implementation process started, what was known before about the NHSSs, feeling about the introduction of the NHSSs).
- What is your experience in implementing the NHSSs?
- What parts (standards) of the NHSSs are you implementing? (Why? How? Who is involved?)
- What support do you have/did you receive to assist in the implementation process? (probe on implementation guidelines, clinical guidelines available, supervision checklist by NDoH etc.; by LHS: workshops, trainings, meetings, supervision checklist, etc.)
- What changes did you experience? (probe on enablers, changes at different levels, national, regional, facility level and self) How do you feel about it?
- What barriers did you encounter? (probe on barriers at different levels) How do you feel about it?
- How do you monitor the implementation plan?
- How can the implementation process move forward? (probe at different levels of LHS management: national, regional, facility level, government and self).
